# Incidence, contributing factors, and predictors of diagnostic errors in medical inpatients: A retrospective cohort study

**DOI:** 10.1002/jhm.70268

**Published:** 2026-02-08

**Authors:** Caterina E. Marx, Elena Hofmann, Martin Perrig, Gurpreet Dhaliwal, Wolf Hautz, Jörg P. Isenegger, Ernst Lipp, Drahomir Aujesky, Manuel R. Blum, Tobias Tritschler

**Affiliations:** ^1^ Department of General Internal Medicine Inselspital, Bern University Hospital, University of Bern Bern Switzerland; ^2^ Department of Medicine University of California San Francisco California USA; ^3^ Medical Service, San Francisco VA Medical Center San Francsico California USA; ^4^ Department of Emergency Medicine Inselspital, Bern University Hospital, University of Bern Bern Switzerland; ^5^ Department of Medicine Hospital Riggisberg Riggisberg Switzerland; ^6^ Department of Medicine Hospital Aarberg Aarberg Switzerland; ^7^ Department of Medicine Hospital Interlaken, Hospitals Frutigen Meiringen Interlaken Interlaken Switzerland; ^8^ Department of Medicine, Ottawa Hospital Research Institute University of Ottawa Ontario Canada

## Abstract

**Background:**

Diagnostic error is a major patient safety concern in hospitals, yet most studies have focused on selected high‐risk subgroups, leaving the broader general internal medicine inpatient population understudied.

**Objectives:**

To determine the incidence, contributing factors, resulting harm, and predictors of diagnostic error in medical inpatients.

**Methods:**

This retrospective cohort study included adults admitted to internal medicine between 01/2022 and 12/2022 at one tertiary and 4 secondary care hospitals in Switzerland. Retrieved admissions were randomly ordered, and electronic medical records were reviewed sequentially by two clinicians using standardized instruments, until reaching a pre‐specified target threshold of 50 patients with ≥1 diagnostic error, enabling analysis of five predictors. The primary outcome was the occurrence of a diagnostic error. The secondary outcome was the resulting level of harm. Five pre‐specified predictors were analyzed using multivariable logistic regression.

**Results:**

Of 347 patients (median age 73 [interquartile range, 61–81] years; 140 [40.3%] female), 52 (15%; 95% confidence interval [CI], 11.6%–19.1%) experienced ≥1 diagnostic error, causing harm in 43/52 patients (82.7%; 95% CI 70.3%–90.6%). The most common contributing factors were failures to consider the correct diagnosis (40/52, 76.9%), order appropriate tests (31/52, 59.6%), and act on critical physical exam findings (30/52, 57.7%). Neurocognitive/psychiatric disorders (odds ratio [OR], 2.20; 95% CI, 1.20–4.10) and active cancer (OR, 2.10; 95% CI, 1.01–4.20) independently predicted diagnostic error.

**Conclusions:**

Diagnostic error is common among adult medical inpatients and causes harm. We identified neurocognitive/psychiatric disorders and active cancer as patient‐level predictors of diagnostic error, providing a basis for future studies on targeted interventions.

## INTRODUCTION

Diagnostic errors are a major patient safety concern, accounting for approximately 10% of patient deaths and 17% of adverse events in hospitals.^1^, ^2^ They are the leading cause of medical malpractice claims and carry significant costs.^1^, ^2^, ^3^ The problem gained widespread attention with the 2015 landmark report *Improving Diagnosis in Healthcare* ^2^ and received renewed emphasis when the World Health Organization made it the 2024 World Patient Safety Day theme.^4^ Despite longstanding efforts, diagnostic errors remain prevalent, affecting 5%–13% of outpatients and 5%–23% of inpatients.^5^, ^6^, ^7^, ^8^, ^9^, ^10^, ^11^, ^12^, ^13^

Prior studies have largely focused on subgroups considered high‐risk for diagnostic error, such as readmissions,^10^ intensive care unit (ICU) admissions,^8^, ^9^ Covid‐19 patients,^12^ or in‐hospital deaths.^8^, ^14^ These studies are informative but limit the generalizability of findings to the broader internal medicine inpatient population. Consequently, the incidence and contributing factors of diagnostic error in general medical inpatients remain incompletely characterized. Such information is essential to inform broadly applicable prevention strategies in routine clinical practice.

Diagnostic errors arise from a complex interplay of cognitive (e.g., misinterpretation of results), system‐related (e.g., test unavailability), and “no‐fault” errors (e.g., atypical disease presentation).^15^ Prior efforts targeting specific cognitive and systemic factors have yielded mixed results.^16^ A recent study found that most diagnostic errors are preventable, underscoring the need for effective preventive measures.^13^ Identifying patient‐level predictors may allow a new approach for targeted prevention.

This retrospective multicenter cohort study aimed to describe the incidence, contributing factors, and resulting harm of diagnostic error in medical inpatients and to identify clinical predictors.

## METHODS

### Study setting and population

We included all adults admitted to general internal medicine departments of one tertiary and 4 secondary care hospitals of a Swiss hospital network (Insel Group) between January 1, 2022 and December 31, 2022. Switzerland has a universal healthcare insurance system. Participating hospitals have dedicated internal medicine units staffed by residents and supervised by internists. Inclusion criteria were age ≥18 years, admission to an internal medicine ward, and general consent for use of health‐related data for research. Patients treated by a reviewer were excluded. For patients with multiple admissions in 2022, only the first admission was analyzed to avoid bias from prior clinical decisions. The study was approved by the Cantonal Ethics Committee Bern, Switzerland (BASEC‐ID: 2023‐01531).

### Data collection

Eligible patients were identified via hospital database search. Patient demographics, active diagnoses, medications, and in‐hospital outcomes (length of stay, admission to intermediate care (IMC) or ICU, readmission, in‐hospital mortality) were extracted automatically (Supporting Information S1: Methods 1). All hospitals used the same electronic medical record system, enabling consistent data extraction. Missing data were manually verified.

### Physician reviewer training

Two experienced internists (CM, EH) were trained to review medical records using the revised Safer Dx tool^17^ and Diagnostic Error and Evaluation Research (DEER) taxonomy^18^ on five patients from morbidity and mortality conferences.

### Identification of diagnostic errors, contributing factors, and harms

Reviewers (CM, EH) independently analyzed medical records using the revised 13‐item Safer Dx tool (Supporting Information S1: Methods 2) to identify diagnostic errors.^17^ This instrument has been widely used in different settings, including medical inpatients.^8^, ^9^, ^10^, ^14^, ^17^, ^19^, ^20^, ^21^ Each item is rated from 1 (*strongly disagree*) to 7 (*strongly agree*). Item #13 determines whether a diagnostic opportunity was missed: ≥5 indicated “diagnostic error,” 3–4 “unsure” and ≤2 “no error.” Disagreement and “unsure” cases were resolved in meetings with senior internists (MB, TT). Other specialists were consulted as needed.

Episodes of care under review included the internal medicine stay, including the emergency department. Temporary stays in other departments were not reviewed. Reviewers followed a chronological approach, starting with information available at admission, including transfer and progress notes, vital signs, all inpatient tests, discharge letters, nursing and physical therapy notes. Post‐discharge records were reviewed if applicable.

For confirmed diagnostic errors, contributing factors were classified using the DEER taxonomy (Supporting Information S1: Methods 3),^18^ which maps failure points in the diagnostic process and has been widely used in clinical settings.^8^, ^10^, ^11^, ^14^, ^20^, ^21^, ^22^ Because a diagnostic error may have several contributing factors, reviewers could select multiple failure points per case. Discrepancies were resolved through discussion or senior input (MB, TT).

The level of harm resulting from a diagnostic error was classified based on a prior definition as “none,” “minor” (i.e., patient inconvenience or dissatisfaction), “moderate” (i.e., short‐term morbidity, increased length of stay, need for higher level of care, or invasive procedure) or “major” (i.e., in‐hospital death, permanent disability, or (near) life‐threatening event) (Supporting Information S1: Methods 4).^23^ For patients with multiple errors, the most severe harm was recorded.

### Clinical predictors of diagnostic error

We evaluated the association between five pre‐specified clinical predictors and diagnostic error. Predictors were selected based on prior literature,^9^, ^11^, ^24^, ^25^ clinical relevance, availability in medical records, and straightforward identification using standardized definitions: (1) Female sex; (2) neurocognitive/psychiatric disorder (i.e., mental illness, dementia, delirium or other cognitive impairment); (3) severe chronic cardiopulmonary, renal or hepatic disease (i.e., heart failure NYHA III/IV, chronic obstructive pulmonary disease GOLD C/D, chronic kidney disease with eGFR <30 mL/min, liver cirrhosis Child‐Pugh class C); (4) immunocompromising conditions (i.e., solid‐organ or hematopoietic cell transplantation or immunosuppressive medication); and (5) active cancer (i.e., under therapy or palliation). Predictors were identified using patient demographics, International Classification of Disease (ICD)‐10 or Anatomical Therapeutic Chemical (ATC) codes (Supporting Information S1: Methods 5).

### Study outcomes

The primary outcome was occurrence of a diagnostic error, defined as a missed opportunity to establish a correct and timely diagnosis, consistent with the revised Safer Dx framework,^17^ and based on a previously published definition of diagnostic errors.^26^, ^27^ The secondary outcome was the resulting level of harm.

### Sample size calculation

Eligible patient records were identified via electronic hospital database query, randomly ordered, and reviewed sequentially until reaching a prespecified threshold of 50 patients with a diagnostic error. This threshold was chosen to allow analysis of five predictors, following the common rule of 10 outcome events per predictor variable,^28^ and for feasibility. Assuming an error rate of 5%–15% based on prior studies,^6^, ^9^, ^10^, ^11^, ^29^ we estimated a minimal sample size of 300 patients.

### Statistical analysis

Patient characteristics and in‐hospital outcomes were compared among patients with and without diagnostic error using chi‐squared, Fisher's exact, and Wilcoxon rank‐sum tests, as appropriate. Diagnostic error incidence, contributing factors and resulting harm were summarized descriptively. 95% Wilson confidence intervals (CI) were calculated. Inter‐rater agreement was assessed using descriptive statistics, Cohen's Kappa, and Gwet's Agreement Coefficient 1 (AC1). Gwet's AC1 was included because it is considered more appropriate than Cohen's Kappa when outcome prevalences are very low or high.^30^ Agreement >0.8 is usually considered almost perfect.^31^

Pre‐specified, unadjusted subgroup analyses for incidence of diagnostic error and resulting harm were conducted according to healthcare setting (tertiary vs. secondary care centers) and multimorbidity (≥3 active conditions). A post‐hoc subgroup analysis was conducted by resuscitation status.

Associations between predictors and diagnostic error were evaluated using multivariable logistic regression, with additional analyses adjusting for age. In a post‐hoc analysis, neurocognitive and psychiatric disorder were modeled as separate predictors. All model variables were tested for interaction.

A two‐sided *p* < 0.05 was considered statistically significant. Analyses were performed using R Statistical Software, version 4.2.0.

## RESULTS

### Patient selection and characteristics

Of 3557 eligible patients, we reviewed 347 cases until reaching the pre‐defined threshold of 50 patients with a diagnostic error. Median age was 73 years (interquartile range [IQR], 61–81) and 140 patients (40.3%) were female (Table 1 and Supporting Information S1: Table 1). 177 patients (51%) were multimorbid. Neurocognitive/psychiatric disorder was present in 145 patients (41.8%), severe chronic cardiopulmonary, renal, or hepatic disease in 53 patients (15.3%), immunocompromising conditions in 68 patients (19.6%), and active cancer in 62 patients (17.9%). Forty patients (11.5%) required IMC/ICU care, 13 patients (3.7%) died, and 58 patients (16.7%) were readmitted within 30 days. The analyzed cohort was representative of the overall eligible population, except for a higher prevalence of neurocognitive/psychiatric disorder (41.8 vs. 34.9%).

**Table 1:** Patient characteristics and in‐hospital outcomes according to the absence or presence of a diagnostic error.

	Diagnostic error absent (*n* = 295)	Diagnostic error present (*n* = 52)	*p* value
**Patient characteristics**			
Age (years), median (IQR)	73 (60–80)	74 (66.7–81)	.32
Female sex, *n* (%)	118 (40)	22 (42.3)	.87
Multimorbidity,^a^ *n* (%)	140 (47.5)	37 (71.2)	<.01
Clinical predictors			
Neurocognitive or psychiatric disorder, *n* (%)	115 (39)	30 (57.7)	.02
Chronic cardio‐pulmonary, renal, or hepatic disease, *n* (%)	43 (14.6)	10 (19.2)	.52
Immunocompromising condition, *n* (%)	58 (19.7)	10 (19.2)	>.9
Active cancer, *n* (%)	48 (16.3)	14 (26.9)	>.9
Number of ICD‐10 codes, median (IQR)	13 (9–19)	19 (14–27)	<.001
Number of ATC codes, median (IQR)	9 (5–13)	14 (7–17)	<.001
Weighted Charlson Comorbidity Index, median (IQR)	2 (1–4)	4 (2–5.3)	<.001
Resuscitation status at admission, *n* (%)^b^			.01
Full code	181 (61.4)	22 (42.3)	
Do not resuscitate	113 (38.3)	30 (57.7)	
Healthcare setting, *n* (%)			.51
Tertiary care center	182 (61.7)	29 (55.8)	
Secondary care center	113 (38.3)	23 (44.2)	
Admission status, *n* (%)			>.99
Elective	28 (9.5)	5 (9.6)	
Emergency	267 (90.5)	47 (90.4)	
Day of admission, *n* (%)			.56
Workday	224 (75.9)	42 (80.8)	
Weekend/holiday	71 (24.1)	10 (19.2)	
Shift at admission, *n* (%)			.59
Dayshift (8 a.m. to 5 p.m.)	121 (41)	24 (46.2)	
Nightshift (5 p.m. to 8 a.m.)	174 (59)	28 (53.8)	
**In‐hospital outcomes**			
Length of stay (days), median (IQR)	5 (3–8)	12.5 (6.7–18)	<.001
Admission to intermediate or intensive care, *n* (%)	29 (9.8)	11 (21.2)	.03
30‐day readmission rate, *n* (%)	48 (16.3)	10 (19.2)	.75
In‐hospital mortality, *n* (%)	9 (3.1)	4 (7.7)	.11

Abbreviations: ATC, anatomical therapeutic chemical; ICD, International Classification of Disease; IQR, interquartile range.

^a^ Multimorbidity was defined as the presence of ≥3 active conditions, based on the weighted Charlson Comorbidity Index.

^b^ There was one missing value for resuscitation status in a patient without diagnostic error.

Seventeen baseline characteristics were missing after automated data abstraction (14 Charlson Comorbidity Index, three resuscitation status), with all but one retrieved after manual chart review.

### Incidence of diagnostic error and level of harm

Diagnostic error occurred in 52 of 347 patients (15%; 95% CI 11.6%–19.1%) with a total of 61 diagnostic errors identified. Eight patients (2.3%) experienced ≥1 diagnostic error. Diagnostic error resulted in harm in 43/52 patients (82.7%; 95% CI 70.3%–90.6%), with major harm in 5/52 patients (9.6%), moderate harm in 30/52 patients (57.7%), and minor harm in 8/52 patients (15.4%) (Supporting Information S1: Table 2). Nine patients (17.3%) experienced no harm. Diagnostic errors most frequently involved missed or delayed diagnoses of infectious (18/61, 29.5%) and cardiovascular diseases (16/61, 26.2%) (Supporting Information S1: Table 3). Summary reports of representative case examples are presented in Table 2.

**Table 2:** Summary reports of representative cases with identified diagnostic error.

Case description	Contributing factors according to DEER taxonomy	Level of harm
A patient was admitted with a 2‐week history of progressive cognitive decline, having previously been independent at home. Two weeks prior to admission, the patient had sustained a fall, prompting a cerebral MRI that revealed microangiopathic changes and possible vascular dementia. In the emergency department, the treating team decided against repeat imaging. The following morning during physiotherapy, two episodes of freezing accompanied by twitching movements were observed. An attempted lumbar puncture was unsuccessful. Over the next 2 days, the patient showed further neurological deterioration, prompting repeat MRI and lumbar puncture. Both tests revealed no explanation for the cognitive decline. An EEG was scheduled for the next day and demonstrated epileptiform potentials. Levetiracetam was initiated with rapid neurological improvement.	Delayed diagnosis of an epilepsy due to – suboptimal weighing of a critical history data – suboptimal weighing of a critical physical exam finding – delay in ordering and performing needed tests – delay in considering the correct diagnosis, too much weight on competing diagnosis and failure to recognize urgency	Moderate
A patient with metastatic pancreatic cancer was admitted with elevated temperature, redness around the ankle, calf pain, and elevated D‐dimer levels. An infection of unclear origin was suspected; antibiotic therapy was withheld. Due to worsening fever and abdominal pain with diffusely tender abdomen on palpation, a thoracoabdominal CT scan was performed 2 days later, revealing pulmonary embolism and ascites. Ascites was partially hyperechoic on ultrasound. Spontaneous bacterial peritonitis was considered, but a malignancy‐associated fever and pain was deemed as more likely. The following day, a significant increase in CRP prompted paracentesis, which confirmed the diagnosis of spontaneous bacterial peritonitis.	Delayed diagnosis of a pulmonary embolism and spontaneous bacterial peritonitis due to – delay in following up on a critical history data – suboptimal weighing and delay in following up on a critical physical exam finding – delay in ordering and performing needed tests – error in clinician interpretation of test – too little consideration given to the correct diagnosis and delay to recognize urgency and complications	Moderate
The patient presented with tachycardia, tachypnea, fever, and a red, swollen leg with hemorrhagic bullae. Erysipelas was diagnosed, and antibiotic therapy was initiated. Due to highly elevated D‐dimer levels, an ultrasound was performed, which ruled out deep vein thrombosis. CT pulmonary angiography could not be performed because of an iodine allergy. A transthoracic echocardiogram revealed no signs of right heart strain. As pulmonary embolism could not be definitively excluded, therapeutic anticoagulation was initiated, and a ventilation‐perfusion scintigraphy was planned. During the night, the patient became increasingly hypotensive, tachycardic, and required supplemental oxygen. The deterioration was suspected to be due to pulmonary embolism. Over the next few hours, the patient exhibited progressive skin changes and pain out of proportion, prompting an emergency surgery for necrotizing fasciitis the following morning.	Delayed diagnosis of sepsis due to necrotizing fasciitis due to – misinterpreted and suboptimal weighing of a critical physical exam finding – too much weight on competing diagnosis, failure in considering the correct diagnosis and failure to recognize urgency	Moderate
The patient was admitted with diarrhea, weight loss, and melena. Colonoscopy revealed a high‐grade stenosis at the rectosigmoid junction, attributed to radiation proctitis from prior radiotherapy for cervical cancer. A repeat colonoscopy with balloon dilation was scheduled. Anticoagulation with rivaroxaban, prescribed for a history of extensive deep venous thrombosis, was paused. Three days later, the patient reported sharp chest pain on inspiration, with oxygen saturation dropping to 93%. The patient's condition deteriorated with increasing dyspnea, tachycardia, hypotension, fever, rising CRP levels and higher oxygen requirements, raising suspicion for pneumonia. However, observation was pursued as the chest x‐ray did not reveal infiltrates. Later that day, the patient experienced syncope. An emergent chest CT scan revealed bilateral central to subsegmental pulmonary embolism.	Delayed diagnosis of pulmonary embolism due to – suboptimal weighing of and failure to follow‐up on critical history data – suboptimal weighing of and failure to follow‐up on critical physical exam finding – delay in ordering needed test – delay in considering the correct diagnosis, too much weight on coexisting diagnosis and failure to recognize complications	Major

Abbreviations: BNP, B‐type natriuretic peptide; CRP, C‐reactive protein; CT, computed tomography; MRI, magnetic resonance imaging; NSTEMI, non‐ST‐elevation myocardial infarction.

Patients with diagnostic error had a longer length of stay (median [IQR], 12.5 [6.7–18] vs. 5 [3–8], *p* < .001), higher number of active ICD‐10 codes (median [IQR], 19 [14–27] vs. 13 [9–19], *p* < .001), higher number of ATC codes (median [IQR], 14 [7–17] vs. 9 [5–13], *p* < .001), and a higher Charlson Comorbidity Index (median [IQR], 4 [2‐5.3] vs. 2 [1–4], *p* < .001) (Table 1). In addition, they were more likely to be admitted to IMC/ICU (21.2% vs 9.8%, *p* = .03). Four of the 11 patients with diagnostic error who were transferred to IMC/ICU were transferred as a direct consequence of the error.

Reviewers agreed on diagnostic error classification in 299/347 patients (86.2%; 279 “no diagnostic error,” 18 “diagnostic error,” 2 “unsure”) and had discrepancies in 48 cases (13.8%; Gwet's AC1 0.84, Cohen's Kappa 0.43). After team discussion, the two “unsure” cases were reclassified as “no diagnostic error.” Of all 48 discrepant cases, 32 were reclassified as “diagnostic error” and 16 as “no diagnostic error.”

### Subgroup analyses

In unadjusted subgroup analyses, diagnostic error incidence and resulting harm did not differ significantly across healthcare settings (Supporting Information S1: Table 4). Diagnostic error was more common in multimorbid than non‐multimorbid patients (37/177, 20.9% vs. 15/170, 8.8%, *p* = .003) and in those with a “Do Not Resuscitate” status compared to those without (30/143, 21% vs. 22/203, 10.8%, *p* = .01), with no significant difference in the resulting level of harm (Supporting Information S1: Table 4).

### Factors contributing to diagnostic error

**Figure 1:**
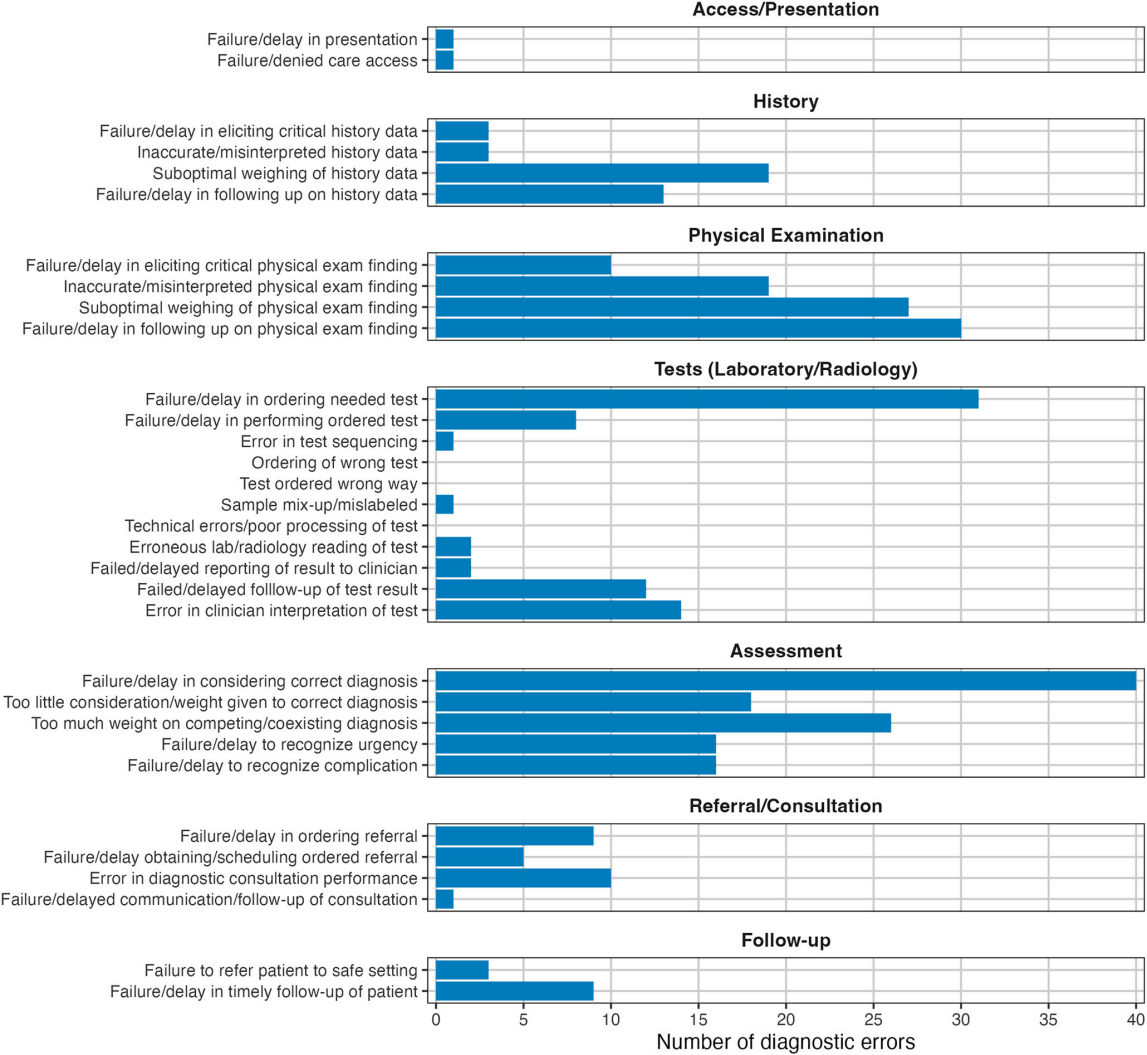
Frequency of contributing factors to diagnostic errors based on the DEER classification. Each horizontal bar represents the number of diagnostic errors associated with a specific failure point in the diagnostic process, as defined by the Diagnostic Error and Evaluation Research (DEER) taxonomy tool. The *x*‐axis indicates how often each contributing factor was involved in a diagnostic error.

Each diagnostic error was related to several contributing factors (mean 6.7 factors per error, standard deviation, 3.04). “Assessment” was the diagnostic process stage most frequently related to diagnostic errors, with “failure/delay in considering the correct diagnosis” identified in 40/52 cases (76.9%), and “too much weight on coexisting/competing diagnosis” in 26/52 cases (50%) (Figure 1). Diagnostic errors were also frequently linked to physical examination and testing, with “failure/delay in ordering needed tests” occurring in 31/52 cases (59.6%), “failure/delay in following up on a critical physical exam finding” in 30/52 cases (57.7%), and “suboptimal weighing of a physical exam finding” in 27/52 cases (51.9%).

### Clinical predictors of diagnostic error

In multivariable analysis, neurocognitive/psychiatric disorder (OR 2.20, 95% CI 1.20–4.10) and active cancer (OR 2.10, 95% CI 1.01–4.20) were independently associated with diagnostic error (Table 3). Additional adjustment for age did not substantially alter the results (Supporting Information S1: Table 5). In a post‐hoc analysis separating neurocognitive and psychiatric disorder as individual predictors, both were independently associated with diagnostic error (neurocognitive disorder: OR 2.02, 95% CI 1.00–3.94; psychiatric disorder: OR 1.99, 95% CI 1.03–3.76) (Supporting Information S1: Table 6). After age adjustment, psychiatric disorder remained an independent predictor (OR 2.23, 95% CI 1.13‐4.38), whereas neurocognitive disorder lost statistical significance (OR 1.84, 95% CI 0.9–3.65). No significant interactions were detected between variables.

**Table 3:** Association between predefined clinical predictors and diagnostic error.

Predictor	Multivariable analysis		
	OR	95% CI	*p* value
Female sex	1.12	0.60−2.06	.72
Neurocognitive or psychiatric disorder	2.20	1.20−4.10	.01
Chronic cardio‐pulmonary, renal or hepatic disease	1.37	0.60−2.91	.43
Immunocompromising condition	0.97	0.42−2.07	.95
Active cancer	2.10	1.01−4.20	.04

*Note*: Multivariable analysis for the association between predefined clinical predictors and diagnostic error, adjusted for all five predictors.

Abbreviations: CI, confidence interval; OR, odds ratio.

## DISCUSSION

This retrospective cohort study found a diagnostic error incidence of 15% among adult medical inpatients, with 83% of affected patients experiencing harm. Errors commonly involved failures to consider the correct diagnosis, order appropriate tests, or act on physical exam findings. Neurocognitive/psychiatric disorders and active cancer were independent predictors of diagnostic error.

Prior US studies have focused on high‐risk populations, such as in‐hospital deaths,^8^ ICU transfers,^8^, ^9^ rapid response team assessments,^11^ and readmissions,^10^ reporting diagnostic error rates of 6%–23%, with 77%–100% of errors resulting in harm.^8^, ^9^, ^10^ We observed a similarly high error rate and harm in our cohort of unselected internal medicine inpatients, suggesting that these patients also constitute a vulnerable population. Notably, our observed error rate exceeds that of a recent US retrospective single‐center study of medical inpatients (7%) using similar methods.^13^ This may reflect our specific review process and broader definition of diagnostic error, including missed diagnostic opportunities in the absence of a final diagnosis.^32^ Overrepresentation of patients with neurocognitive/psychiatric disorder may have further contributed.

Consistent with previous studies using the DEER taxonomy, testing and assessment,^8^, ^10^, ^22^, ^23^, ^33^ including “failure in ordering needed tests,” “failure in considering the correct diagnosis,” and “too much weight on competing diagnosis” were key contributing factors to diagnostic error.^10^, ^21^, ^22^, ^23^ Additionally, our study highlighted issues related to suboptimal weighing and inadequate follow‐up of physical exam findings, underscoring the importance of integrating all clinical findings when formulating a diagnosis.

To our knowledge, few studies have systematically examined pre‐specified patient‐level predictors of diagnostic error.^34^, ^35^ Our results indicate that neurocognitive/psychiatric disorders increase the risk for diagnostic error, possibly due to behaviors that increase physicians' cognitive load, contributing to misdiagnosis,^36^, ^37^ and challenges in communication, such as difficulty obtaining accurate medical history or assessing changes in a patient's condition. Patients with active cancer were also at increased risk for diagnostic error. Autopsy studies have reported discrepancies between premortem and postmortem diagnoses in cancer patients,^25^, ^38^ indicating that diagnostic challenges are common in this population. Clinicians may unintentionally deprioritize or misattribute new symptoms, assuming they are related to the malignancy or treatment. In palliative settings, a focus on comfort measures might further reduce diagnostic vigilance.

The high incidence of diagnostic error in this and previous studies highlights the need for effective interventions. Various approaches have been suggested but have shown limited effects in vignette or observational studies.^16^, ^39^ Clinical trials evaluating their effectiveness are largely absent. The Agency for Healthcare for Research and Quality has developed several tools such as the *Toolkit for Engaging Patients to Improve Diagnostic Safety*, targeting miscommunication by helping patients clearly express health concerns.^40^ This tool could be applied to groups at higher risk of miscommunication, such as patients with neurocognitive or psychiatric disorder, or in time‐pressured settings (e.g., emergency departments), where obtaining an accurate history is critical for diagnosis. The tool *Calibrate Dx* aims to enhance clinicians' knowledge through self‐reflective diagnostic performance analysis,^41^ and targets errors related to incomplete knowledge synthesis, common contributing factors identified in our study. Emerging evidence suggests that the organization of knowledge critically influences diagnostic accuracy.^42^, ^43^ Collaborative knowledge structures such as team‐based discussions facilitate clinical reasoning and help bridge individual knowledge gaps, particularly in complex cases requiring specialized expertise. Building on this, artificial intelligence (AI) and large language models (LLMs) offer potential by supporting clinical reasoning interactively and reducing cognitive load through structured data synthesis.^44^ In a recent retrospective study, LLMs had high diagnostic accuracy when presented with real‐life cases.^45^ However, integration of AI into clinical workflows is still evolving: a recent cluster‐randomized trial evaluating an AI‐driven diagnostic decision support tool generating differential diagnosis lists in emergency patients found no improvement in diagnostic quality.^46^ Similarly, LLMs did not improve physicians' diagnostic reasoning on case vignettes, although the model alone achieved higher diagnostic accuracy.^47^ To gain acceptance, LLMs must integrate seamlessly into workflows and complement natural clinical reasoning. Embedded responsibly in clinical information systems, they may improve diagnostic accuracy, for example, by highlighting symptoms not considered in clinical reasoning, addressing key contributors to diagnostic errors. Identifying patient‐level predictors of diagnostic error enables targeted interventions such as electronic medical record alerts prompting diagnostic time‐outs or team reviews for high‐risk patients. Future work should validate these predictors and prospectively evaluate whether such interventions reduce errors.

Strengths of our study include a broad, randomly selected inpatient sample across various levels of healthcare. The Swiss hospital setting is comparable to the United States, with similar internal medicine staffing models and diagnostic reasoning challenges. Independent chart review by two experienced physicians using validated instruments ensured standardized assessment of medical charts. Team discussions added a layer of quality control, further enhancing reliability of evaluations. With only one missing value, data abstraction was nearly complete. Our standardized review of medical records provides a methodological model for future diagnostic error research.

### Limitations

Our study has several limitations. First, the retrospective approach is susceptible to hindsight bias. To mitigate this, we used a chronological chart review. Second, analysis was limited to follow‐up data within participating hospitals, complicating identification of diagnostic errors when follow‐up occurred in external settings. Also, long‐term harm may have occurred beyond our observation window. Third, we did not assess diagnostic errors in other specialties, potentially overlooking errors during care transitions. Fourth, contributing factors could only be identified when documented, limiting detection of cognitive, communication, or system‐level failures. We also did not evaluate predictors beyond patient factors, such as physician characteristics (e.g., experience) or system factors (e.g., institutional processes), due to the complexity of clinical care involving multiple clinicians in a patient's management, preventing reliable retrospective assessment at an individual level, and because system factors are difficult to capture in a retrospective design limited to the information available in medical charts. Future studies could complement our findings by prospectively examining clinician and system‐level contributors to diagnostic error. Additionally, although patients with diagnostic error had longer hospitalizations, we did not assess directionality of this complex relationship; errors may prolong hospitalization, but longer stays may also increase the risk of error. Fifth, overrepresentation of patients with neurocognitive/psychiatric disorder may have led to an overestimation of diagnostic error incidence. Sixth, about 50% of patients admitted to our hospital network give general consent, which may affect generalizability. Finally, the adequacy of reviewer training was not formally tested, which could have influenced diagnostic error detection.

## CONCLUSION

Diagnostic error is common among medical inpatients and associated with substantial harm. Errors were frequently rooted in failures to consider the correct diagnosis, order appropriate tests, or act based on physical exam findings. Neurocognitive/psychiatric disorders and active cancer emerged as predictors of diagnostic error. Our findings suggest that clinicians should maintain heightened diagnostic vigilance in these at‐risk populations and systematically reassess diagnostic assumptions when certain findings or the treatment response are inconsistent with expectations. Further studies are required to investigate whether targeted interventions for these at‐risk patients can reduce diagnostic error.

## CONFLICT OF INTEREST STATEMENT

The authors declare no conflicts of interest.

## Supporting information

SupplementaryFile_Marx_clean.

## Data Availability

Data will be shared upon reasonable request. C.E. Marx had full access to all the data in the study and takes responsibility for the integrity of the data and the accuracy of the data analysis. C.E. Marx conducted and is responsible for the data analysis.
