# Supplementary material for: Incidence, contributing factors, and predictors of diagnostic errors in medical inpatients: A retrospective cohort study
Source: J Hosp Med. 2026 Feb 8;21(8):854–63. doi: 10.1002/jhm.70268 (PMC13432594; doi:10.1002/jhm.70268)
Supplement: Supplementary file 1 — SupplementaryFile_Marx_clean. [file JHM-21-854-s001.docx]

**Supplementary File**

Methods 1. List of variables abstracted for data collection p. 2

Methods 2. Instructions for reviewers on using the revised SaferDx Tool p. 3

Methods 3. Instructions for reviewers on using the Diagnostic Error and Evaluation Research

(DEER) Taxonomy p. 5

Methods 4. Instructions for reviewers on defining level of harm p. 6

Methods 5. Definition of pre-selected predictors p. 7

Supplementary Table 1: Patient characteristics of all eligible patients and the reviewed cohort p. 8

Supplementary Table 2: Types of harm resulting from diagnostic errors p. 9

Supplementary Table 3: Missed or delayed diagnoses by disease category p. 10

Supplementary Table 4: Subgroup analyses p. 12

Supplementary Table 5: Multivariable analysis: association between predefined clinical

predictors and diagnostic error, adjusted for age p. 13

Supplementary Table 6: Post-hoc multivariable analysis including neurocognitive and psychiatric disorders as separate predictors p. 14

References p. 15

**Methods 1.** List of variables abstracted for data collection

| **Variable** | **Data Source** |
| --- | --- |
| **Age** | Age at admission^a^ |
| **Sex** | Gender^a^ |
| **Number and type of ICD 10-codes** | Number and specific ICD 10-codes of all coded diagnoses based on the discharge report of the hospitalization under analysis^a^ |
| **Number and type of ATC-codes** | Number and specific ATC-codes of all coded medication based on the discharge report of the hospitalization under analysis^a^ |
| **Weighted Charlson Comorbidity Index** | Based on ICD 10-codes, calculated with R package “comorbidity” |
| **Resuscitation status** | First resuscitation status documented at admission^a^ |
| **Healthcare setting** | Admission to secondary or tertiary hospital^a^ |
| **Admission status** | Elective or emergency admission^a^ |
| **Day of admission** | Time of admission to the ward on working days (Monday-Friday) or at the weekend (Saturday-Sunday) including public holidays^a^ |
| **Shift at admission** | Time of admission to the ward during dayshift (08.00 a.m. – 05.00 p.m.) or nightshift (05.00 p.m. – 08.00 a.m.) ^a^ |
| **Length of stay** | Difference between time of discharge and time of admission in hours, divided by 24^a^ |
| **Admission to intermediate or intensive care** | Admission to an intermediate or intensive care department during the hospitalization under analysis^a^ |
| **30-day readmission rate** | Readmission to the Insel Group within 30 days after discharge^a^ |
| **In-hospital mortality** | Death during hospitalization^a^ |

^a^ automatically extracted from electronic health records

**Methods 2.** Instructions for reviewers on using the revised SaferDx Tool ^1^

Instructions: The revised SaferDx Tool consists of questions that are intended to identify missed opportunities in diagnosis. The final question (no 13) summarizes the overall impression of diagnostic error after systematically considering the individual items. A high rating most likely represents a diagnostic error, and a low rating indicates that no error was identified. For a question that does not apply for the specific case under review, the reviewer should record option 1 (for example, for question 4, if there are no alarm symptoms or “Red Flags” to begin with, record 1 for the item and move to the next question).

Examples: If a patient with a history of i.v. drug use is admitted to the hospital with a week of back pain and fever, it should be considered as a missed opportunity if MRI of the spine was not considered. No missed opportunity would be determined when a young, healthy patient with 2 days of dry cough and no other associated signs and symptoms was treated with watchful waiting even if she returned in 5 days with fever and a subsequent chest x-ray showed community-acquired pneumonia.

*Rate the following items for the episode of care under review*^a^ *on a scale from 1 to 7:*

*1 – 2 – 3 – 4 – 5 – 6 - 7 (1 = strongly disagree, 4 = neutral, 7 = strongly agree)*

^a^ *episode of care = the entire length of the hospital stay including the emergency room visit that led to the hospital admission. If the patient was transferred to the ICU/IMCU, consult the transfer report. The stay in the ICU/IMCU will not be reviewed.*

|  | **Item** | **Score** |
| --- | --- | --- |
| 1. | The documented history was suggestive of an alternate diagnosis, which was not considered in the diagnostic process. |  |
| 2. | The documented physical exam (including vital signs) was suggestive of an alternate diagnosis, which was not considered in the diagnostic process. |  |
| 3. | Data gathering through history, physical exam, and review of prior documentation (including prior laboratory, radiology, pathology or other results) was incomplete, given the patient’s medical history and clinical presentation. |  |
| 4. | Alarm symptoms or “Red Flags” (i.e. features in the clinical presentation that are considered to predict serious disease) were not acted upon. |  |
| 5. | The diagnostic process was affected by incomplete or incorrect clinical information given to the care team by the patient or their primary caregiver. |  |
| 6. | The clinical information (i.e. history, physical exam or diagnostic data) should have prompted additional diagnostic evaluation through tests or consults. |  |
| 7. | The diagnostic reasoning was not appropriate, given the patient’s medical history and clinical presentation. |  |
| 8. | Diagnostic data (laboratory, radiology, pathology or other results) available or documented were misinterpreted in relation to the subsequent final diagnosis. |  |
| 9. | There was missed follow-up of available or documented diagnostic data (laboratory, radiology, pathology or other results) in relation to the subsequent final diagnosis. |  |
| 10. | The differential diagnosis was not documented OR The documented differential diagnosis did not include the subsequent final diagnosis. |  |
| 11. | The final diagnosis was not an evolution of the care team’s initial presumed diagnosis (or working diagnosis). |  |
| 12. | The clinical presentation at the initial or subsequent presentation was mostly typical of the final diagnosis. |  |
| 13. | In conclusion, based on all the above questions, the episode of care under review has a missed opportunity to make a correct and timely diagnosis. |  |

*Additional information:*

|  | **Item** | **yes/no** |
| --- | --- | --- |
| 1. | Care episode involves a management error.^b^ |  |
| 2. | Care escalation (e.g. transfer to hospital with higher level of care, IMCU or ICU) was related to worsening of an original correctly diagnosed condition that the patient initially presented with (rather than from something being missed initially) |  |
| 3. | Patient initially refused admission or additional evaluation. |  |

^b^ Regardless of the presence of a diagnostic error, the care episode can involve a management error (= incorrect therapy or management after a correct diagnosis). For instance, a patient may present with a fracture that was diagnosed correctly but was treated with the wrong intervention.

*Brief description of missed diagnostic opportunity or management error and any relevant thoughts and observations that helped with your decision (for or against):*

**Methods 3.** Instructions for reviewers on using the Diagnostic Error and Evaluation Research (DEER) Taxonomy ^2^

Instructions: If question 13 of the revised SaferDx tool is scored ≥ 5 points, this is considered as a diagnostic error. Continue with the DEER tool to characterize the error type. As a diagnostic error may have several contributing factors, you may select more than one sub-item for each diagnostic error.

| **Where in the diagnostic process?** | | **What went wrong?** |
| --- | --- | --- |
| 1. Access/Presentation | **A** | Failure/delay in presentation |
|  | **B** | Failure/denied care access |
| 2. History | **A** | Failure/delay in eliciting a critical piece of history data |
|  | **B** | Inaccurate/misinterpreted critical piece of history data |
|  | **C** | Suboptimal weighing of a critical piece of history data |
|  | **D** | Failure/delay to follow-up on a critical piece of history data |
| 3. Physical Examination | **A** | Failure/delay in eliciting a critical physical exam finding |
|  | **B** | Inaccurate/misinterpreted critical physical exam finding |
|  | **C** | Suboptimal weighing of a critical physical exam finding |
|  | **D** | Failure/delay in following up on a critical physical exam finding |
| 4. Tests | *Ordering* | |
| (Laboratory/Radiology) | **A** | Failure/delay in ordering needed test(s) |
|  | **B** | Failure/delay in performing ordered test(s) |
|  | **C** | Error in test sequencing |
|  | **D** | Ordering of wrong test(s) |
|  | **E** | Test ordered wrong way |
|  | *Performance* | |
|  | **F** | Sample mix-up/mislabeled (e.g., wrong patient/test) |
|  | **G** | Technical errors/poor processing of specimen/test |
|  | **H** | Erroneous lab/radiology reading of test |
|  | **I** | Failed/delayed reporting of result to clinician |
|  | *Clinician Processing* | |
|  | **J** | Failed/delayed follow-up of (abnormal) test result |
|  | **K** | Error in clinician interpretation of test |
| 5. Assessment | *Hypothesis Generation* | |
|  | **A** | Failure/delay in considering the correct diagnosis |
|  | *Suboptimal Weighing/Prioritizing* | |
|  | **B** | Too little consideration/weight given to the correct diagnosis |
|  | **C** | Too much weight on competing/coexisting diagnosis |
|  | *Recognizing Urgency/Complications* | |
|  | **D** | Failure/delay to recognize urgency |
|  | **E** | Failure/delay to recognize complication(s) |
| 6. Referral/Consultation | **A** | Failure/delay in ordering referral |
|  | **B** | Failure/delay obtaining/scheduling ordered referral |
|  | **C** | Error in diagnostic consultation performance |
|  | **D** | Failure/delayed communication/follow-up of consultation |
| 7. Follow-up | **A** | Failure to refer patient to close/safe setting/monitoring |
|  | **B** | Failure/delay in timely follow-up/rechecking of patient |

**Methods 4.** Instructions for reviewers on defining level of harm

Instructions: When a diagnostic error has been confirmed, determine the level of harm that has occurred.

| A | No harm |
| --- | --- |
| B | Minor harm: patient inconvenience or dissatisfaction |
| C | Moderate harm: short-term morbidity, increased length of stay, need for higher level of care or invasive procedure |
| D | Major harm: in-hospital death, permanent disability or (near) life-threatening event |

**Methods 5.** Definition of pre-selected predictors

| Predictor | Definition | Identification in Database |
| --- | --- | --- |
| Female sex | Female sex | Computerized data extraction |
| Neurocognitive or psychiatric disorder | Mental illness, dementia, delirium or other cognitive impairment | ICD10-Codes^a^: F00 - F48, F50, F60 – F99, G30 – G31, G80, G93.4, G94.3, U51.1, U51.2 |
| Severe chronic cardiopulmonary, renal or hepatic disease | Congestive heart failure NYHA III or IV, chronic obstructive pulmonary disease GOLD C or D, severe chronic kidney disease (eGFR <30 ml/min), liver cirrhosis Child-Pugh class C | ICD10-Codes^a^: I50.04, I50.05, I50.13, I50.14, J44.01, J44.00, J44.10, J44.11, J44.80, J44.81, J44.90, J44.91, N18.4, N18.5, K74.72 |
| Immunocompromising  condition | Solid-organ or hematopoietic cell transplantation, immunosuppressive medications | ICD10-Codes^a^: Z94.0, Z94.1, Z94.2, Z94.3, Z94.4, Z94.8, D90  ATC-Codes: L01, L04, H02AB, H02B |
| Active cancer | Cancer under therapy or palliation | ICD10-Codes^a^: Z92.3, Z92.6, C00-C97 |

Abbreviations: ATC, Anatomical Therapeutic Chemical Classification; ICD10, International Classification of Disease; IDSC, Insel Data Science Center

^a^ ICD10 GM, Version 2024 was used for identification of all ICD-Codes

**Supplementary Table 1:** Patient characteristics of all eligible patients and the reviewed cohort

|  | **Eligible Patients**  **(n = 3557)** | **Cases Not Reviewed**  **(n = 3210)** | **Cases Reviewed**  **(n = 347)** |
| --- | --- | --- | --- |
| **Patient Characteristics** |  |  |  |
| Age (years), median (IQR) | 68 (60-80) | 72 (60-80) | 73 (61 - 81) |
| Female sex, n (%) | 1492 (41.9) | 1352 (42.1) | 140 (40.3) |
| Multimorbidity^a^, n (%) | 1633 (45.9) | 1456 (45.4) | 177 (51) |
| Clinical predictors |  |  |  |
| Neurocognitive or psychiatric disorder, n (%) | 1242 (34.9) | 1097 (34.2) | 145 (41.8) |
| Chronic cardio-pulmonary, renal or hepatic  disease, n (%) | 543 (15.3) | 490 (15.3) | 53 (15.3) |
| Immunocompromising condition, n (%) | 714 (20.1) | 646 (20.1) | 68 (19.6) |
| Active cancer, n (%) | 693 (19.5) | 631 (19.7) | 62 (17.9) |
| Number of ICD-10 codes, median (IQR) | 12 (8-18) | 12 (8-18) | 14 (9-20) |
| Number of ATC codes, median (IQR) | 8 (5-12) | 8 (5-12) | 10 (6-14) |
| Weighted Charlson Comorbidity Index, median (IQR) | 2 (1-4) | 2 (1-4) | 3 (1-4) |
| Resuscitation status at admission^a^, n (%) |  |  |  |
| Full Code | 1974 (55.5) | 1771 (55.2) | 203 (58.5) |
| Do Not Resuscitate | 1390 (39.1) | 1247 (38.8) | 143 (41.2) |
| Healthcare setting, n (%) |  |  |  |
| Tertiary care center | 2144 (60) | 1933 (60.2) | 211 (60.8) |
| Secondary care center | 1413 (39.7) | 1277 (39.8) | 136 (39.2) |
| Admission status^a^, n (%) |  |  |  |
| Elective | 296 (8.3) | 263 (8.2) | 33 (9.5) |
| Emergency | 3259 (91.6) | 2945 (91.7) | 314 (90.5) |
| Day of admission, n (%) |  |  |  |
| Workday | 2814 (79.1) | 2548 (79.4) | 266 (76.7) |
| Weekend/holiday | 743 (20.9) | 662 (20.6) | 81 (23.3) |
| Shift at admission, n (%) |  |  |  |
| Dayshift (8 a.m. to 5 p.m.) | 1500 (42.2) | 1355 (42.2) | 145 (41.8) |
| Nightshift (5 p.m. to 8 a.m.) | 2057 (57.8) | 1855 (57.8) | 202 (58.2) |

Abbreviations: ATC, Anatomical Therapeutic Chemical; ICD, International Classification of Disease; IQR, interquartile range.

^a^ There were 176 missing values for multimorbidity, 193 missing values for resuscitation status and 2 missing values for admission status in all eligible patients. For the reviewed cases, missing values were manually added after chart review except for one missing value for resuscitation status.

**Supplementary Table 2.** Types of harm resulting from diagnostic errors^a^

| **Level of Harm** | **Number of Patients**  **(n = 52)** |
| --- | --- |
| **No harm** | **9** |
| **Minor harm** | **8** |
| Patient dissatisfaction | 1 |
| Patient inconvenience | 7 |
| **Moderate harm** | **30** |
| Short-term morbidity | 14 |
| Increased length of stay | 9 |
| Need for higher level of care | 3 |
| Need for invasive procedure | 4 |
| **Major harm** | **5** |
| In-hospital death | 2 |
| (Near) life-threatening event | 3 |

^a^ A diagnostic error could result in several types of harm (e.g., need for higher level of care and increased length of stay). In such cases, the most serious resulting harm considered most consequential for the patient was recorded in the table.

**Supplementary Table 3.** Missed or Delayed Diagnoses by Disease Category

| **Missed or Delayed Diagnoses by Disease Category** | **Number of Diagnostic Errors (n=61)** |
| --- | --- |
| **Infectious Diseases, n (%)** | **18 (29.5)** |
| Abscess | 4 |
| Spontaneous bacterial peritonitis | 2 |
| COVID-19 infection | 2 |
| Colitis | 2 |
| Sepsis due to necrotizing fascitis | 1 |
| Epidural abscess | 1 |
| Urinary tract infection | 1 |
| Pleural empyema | 1 |
| Wound infection | 1 |
| Obstructive pyelonephritis | 1 |
| Erysipelas | 1 |
| Endocarditis | 1 |
| **Cardiovascular Diseases, n (%)** | **16 (26.2)** |
| Heart failure | 4 |
| Pulmonary edema | 2 |
| Pulmonary embolism | 2 |
| Hemodynamically significant pericardial effusion | 2 |
| Catheter-associated thrombosis | 1 |
| Hypertensive urgency | 1 |
| Cardiac syncope | 1 |
| Deep venous thrombosis | 1 |
| Coronary artery disease | 1 |
| Severe peripheral artery disease | 1 |
| **Neurological Diseases, n (%)** | **6 (9.8)** |
| Cauda equina syndrome | 1 |
| Encephalitis | 1 |
| Epilepsy | 1 |
| Personality disorder | 1 |
| Stroke | 1 |
| Subarachnoid hemorrhage | 1 |
| **Rheumatological/Musculosceletal Diseases, n (%)** | **6 (9.8)** |
| Arthritis | 3 |
| Large-vessel vasculitis | 2 |
| Fracture | 1 |
| **Neoplastic Diseases, n (%)** | **5 (8.2)** |
| Gastrointestinal tumor | 1 |
| Cancer of unknown etiology | 1 |
| Metastatic melanoma | 1 |
| Lung carcinoma | 1 |
| Renal cell carcinoma | 1 |
| **Bleeding Disorders, n (%)** | **4 (6.6)** |
| Postinterventional bleeding | 2 |
| Hematoma with active bleeding | 1 |
| Active tumor bleeding | 1 |
| **Metabolic Diseases, n (%)** | **3 (4.9)** |
| Symptomatic hyponatremia | 1 |
| Hyperparathyroidism | 1 |
| Acute kidney failure | 1 |
| **Respiratory Diseases, n (%)** | **2 (3.3)** |
| Exacerbation of chronic obstructive pulmonary disease | 1 |
| Organizing pneumonia | 1 |
| **Dermatological Diseases, n (%)** | **1 (1.6)** |
| Drug-induced exanthema | 1 |

**Supplementary Table 4.** Subgroup analyses

| **Outcome** | **Secondary Care Center (n = 136)** | **Tertiary Care Center  (n = 211)** | ***P*-value** |
| --- | --- | --- | --- |
| Diagnostic errors, n (%) | 23 (16.9) | 29 (13.7) | .51 |
| Level of harm |  |  | .92 |
| - Major harm | 2 (8.7) | 3 (10.3) |  |
| - Moderate harm | 13 (56.5) | 17 (58.6) |  |
| - Minor harm | 3 (13) | 5 (17.2) |  |
| - No harm | 5 (21.7) | 4 (13.8) |  |

| **Outcome** | **No Multimorbidity**  **(n = 170)** | **Multimorbidity**  **(n = 177)** | ***P*-value** |
| --- | --- | --- | --- |
| Diagnostic errors, n (%) | 15 (8.8) | 37 (20.9) | **.003** |
| Level of harm |  |  | .57 |
| - Major harm | 1 (6.7) | 4 (10.8) |  |
| - Moderate harm | 7 (46.7) | 23 (62.2) |  |
| - Minor harm | 3 (20) | 5 (13.5) |  |
| - No harm | 4 (26.7) | 5 (13.5) |  |

| **Outcome** | **Resuscitation Status "Full Code”**  **(n = 203)** | **Resuscitation Status "Do Not Resuscitate” (n = 143)** | ***P*-value** |
| --- | --- | --- | --- |
| Diagnostic errors, n (%) | 22 (10.8) | 30 (21) | **0.01** |
| Level of harm |  |  | 0.48 |
| - Major harm | 1 (4.5) | 4 (13.3) |  |
| - Moderate harm | 13 (59.1) | 17 (56.7) |  |
| - Minor harm | 5 (22.7) | 3 (10) |  |
| - No harm | 3 (13.6) | 6 (20) |  |

**Supplementary Table 5.** Multivariable analysis: association between predefined clinical predictors and diagnostic error, adjusted for age

|  |  | **Multivariable Analysis  (age-adjusted)** | | |
| --- | --- | --- | --- | --- |
| **Predictor** |  | OR | 95% CI | *P* value |
| Female sex |  | 1.15 | 0.62 - 2.13 | .65 |
| Neurocognitive or  psychiatric disorder |  | 2.24 | 1.22 - 4.19 | **.01** |
| Severe chronic cardio-pulmonary, renal or hepatic disease |  | 1.26 | 0.54 - 2.71 | .57 |
| Immunocompromising  condition |  | 0.97 | 0.42 - 2.07 | >.9 |
| Active cancer |  | 2.04 | 0.98 - 4.10 | **.049** |

Abbreviations: CI, confidence interval; OR, odds ratio

**Supplementary Table 6.** Post-hoc multivariable analysis including neurocognitive and psychiatric disorders as separate predictors

|  |  | **Multivariable Analysis** | | |  | **Multivariable Analysis (adjusted for age)** | | |
| --- | --- | --- | --- | --- | --- | --- | --- | --- |
| **Predictor** |  | OR | 95% CI | *P* value |  | OR | 95% CI | *P* value |
| Female sex |  | 1.13 | 0.60 - 2.08 | .70 |  | 1.15 | 0.62 - 2.13 | .65 |
| Neurocognitive disorder |  | 2.02 | 1.00 - 3.94 | **.04** |  | 1.84 | 0.90 - 3.65 | **.09** |
| Psychiatric disorder |  | 1.99 | 1.03 – 3.76 | **.04** |  | 2.23 | 1.13 – 4.38 | **.02** |
| Chronic cardio-pulmonary,  renal or hepatic disease |  | 1.34 | 0.58 - 2.86 | .47 |  | 1.23 | 0.53 - 2.66 | .62 |
| Immunocompromising  condition |  | 1.00 | 0.44 – 2.13 | >.99 |  | 0.99 | 0.43 - 2.12 | .99 |
| Active cancer |  | 2.11 | 1.02 – 4.24 | **.04** |  | 2.06 | 0.99 - 4.14 | .05 |
| Age |  | - | - | - |  | 1.01 | 0.99 - 1.04 | .25 |

Post-hoc multivariable analysis for the association between predefined clinical predictors and diagnostic error including neurocognitive and psychiatric disorders as separate predictors, adjusted for all predictors. Neurocognitive disorder includes the ICD-10 codes F00-F09, G30-G31, G80, G93.4, G94.3, U51.1, U51.2. Psychiatric disorder includes the ICD-10 codes *F10-F48, F50, F60-F99*.

Abbreviations: CI, confidence interval; OR, odds ratio

**References**

1. Singh H, Khanna A, Spitzmueller C, Meyer AND. Recommendations for using the Revised Safer Dx Instrument to help measure and improve diagnostic safety. *Diagnosis*. 2019;6(4):315-323. doi:10.1515/dx-2019-0012

2. Schiff GD, Kim S, Abrams R, et al. Diagnosing Diagnosis Errors: Lessons from a Multi-institutional Collaborative Project. In: Henriksen K, Battles JB, Marks ES, Lewin DI, editors. Advances in Patient Safety: From Research to Implementation (Volume 2: Concepts and Methodology). Rockville (MD): Agency for Healthcare Research and Quality (US); 2005 Feb.
